# Paternal phylogeographic structure of the brown bear (*Ursus arctos*) in northeastern Asia and the effect of male-mediated gene flow to insular populations

**DOI:** 10.1186/s40851-017-0084-5

**Published:** 2017-11-30

**Authors:** Daisuke Hirata, Tsutomu Mano, Alexei V. Abramov, Gennady F. Baryshnikov, Pavel A. Kosintsev, Koichi Murata, Ryuichi Masuda

**Affiliations:** 1 Department of Biological Sciences, Faculty of Science, Hokkaido University, Sapporo, 060-0810 Japan; 2 Institute of Environmental Sciences, Hokkaido Research Organization, Sapporo, 080-0819 Japan; 3 Zoological Institute, Russian Academy of Sciences, St. Petersburg, 199034 Russia; 4 Institute of Plant and Animal Ecology, Russian Academy of Sciences, Ekaterinburg, 620219 Russia; 5 College of Bioresource Sciences, Nihon University, Fujisawa, 252-0880 Japan

**Keywords:** Brown bear, Hokkaido, Phylogeography, Sex-biased dispersal, *Ursus arctos*, Y-chromosomal DNA

## Abstract

**Background:**

Sex-biased dispersal is widespread among mammals, including the brown bear (*Ursus arctos*). Previous phylogeographic studies of the brown bear based on maternally inherited mitochondrial DNA have shown intraspecific genetic structuring around the northern hemisphere. The brown bears on Hokkaido Island, northern Japan, comprise three distinct maternal lineages that presumably immigrated to the island from the continent in three different periods. Here, we investigate the paternal genetic structure across northeastern Asia and assess the connectivity among and within intraspecific populations in terms of male-mediated gene flow.

**Results:**

We analyzed paternally inherited Y-chromosomal DNA sequence data and Y-linked microsatellite data of 124 brown bears from Hokkaido, the southern Kuril Islands (Kunashiri and Etorofu), Sakhalin, and continental Eurasia (Kamchatka Peninsula, Ural Mountains, European Russia, and Tibet). The Hokkaido brown bear population is paternally differentiated from, and lacked recent genetic connectivity with, the continental Eurasian and North American populations. We detected weak spatial genetic structuring of the paternal lineages on Hokkaido, which may have arisen through male-mediated gene flow among natal populations. In addition, our results suggest that the different dispersal patterns between male and female brown bears, combined with the founder effect and subsequent genetic drift, contributed to the makeup of the Etorofu Island population, in which the maternal and paternal lineages show different origins.

**Conclusions:**

Brown bears on Hokkaido and the adjacent southern Kuril Islands experienced different maternal and paternal evolutionary histories. Our results indicate that sex-biased dispersal has played a significant role in the evolutionary history of the brown bear in continental populations and in peripheral insular populations, such as on Hokkaido, the southern Kuril Islands, and Sakhalin.

**Electronic supplementary material:**

The online version of this article (10.1186/s40851-017-0084-5) contains supplementary material, which is available to authorized users.

## Background

Molecular studies based on both biparentally and uniparentally inherited, sex-specific genetic markers have shown empirically that sex-biased dispersal is widespread among mammals, but varies widely in direction and intensity [1–3]. Most mammals show male-biased dispersal (males disperse from their natal area) and female philopatry (females stay in the natal area) [4–6]. Sexually incongruent patterns of genetic differentiation resulting from sex-biased migration are known from diverse taxonomic groups of large mammals, including humans (*Homo sapiens*) [7, 8], bonobos (*Pan paniscus*) [9], chimpanzees (*Pan troglodytes*) [10], orangutans (*Pongo spp.*) [11, 12], hamadryas baboon (*Papio hamadryas hamadryas*) [13], canids (*Canis lupus*) [14, 15], and domesticated animals, such as sheep (*Ovis spp.*) and horses (*Equus caballus*) [16, 17]. The brown bear (*Ursus arctos*), which is widely distributed throughout the Holarctic region, shows strongly male-biased dispersal [18–21], and sex-biased dispersal has significantly influenced the molecular evolutionary history of ursids in general [22].

Some previous phylogeographic studies of the brown bear based on maternally inherited mitochondrial DNA (mtDNA) showed extensive intraspecific geographical genetic structuring in maternal lineages [23–27]. The brown bear population on Hokkaido Island, northern Japan, an insular population peripheral to the Eurasian Continent, is composed of three distinct allopatrically distributed mtDNA lineages [25, 28]. These three maternal lineages apparently diverged on the Eurasian Continent prior to migration onto Hokkaido via land bridges in three different glacial periods, with a southern Hokkaido lineage having colonized first, followed by eastern and then central Hokkaido lineages.

While Hokkaido Island (77,984 km^2^) is much smaller than the adjacent continent, the maternal genetic diversity is higher there than in northern continental Eurasia, where the brown bear predominantly shows a single mtDNA lineage [26, 29–31]. The high level of diversity on Hokkaido is a consequence of the multiple lineages that originated allopatrically and remained allopatric on Hokkaido through female philopatry.

In addition to using maternally inherited mtDNA, the influence of sex-biased gene flow can be measured by using sex-linked markers in the male-specific, non-recombining region of the Y-chromosome [32–36]. In contrast to the genetic structuring evident in brown bear mtDNA, Y-chromosomal DNA shows low intraspecific variation, with no clear phylogeographic structure throughout the Holarctic region [34]. Extensive male-biased dispersal is thought to have resulted in gene flow across large geographical distances and between Asia and the North America, tending to homogenize genetic variation.

The role of male-mediated gene flow in the evolutionary history of brown bears seems to depend on the time interval and distributional area under consideration, and the presence or absence of previous occupant populations. Male-mediated gene flow could have connected bear populations of the Alaskan ABC islands with those of the North American mainland, and played a substantial role in maintaining high genetic variation in insular populations [34]. On the other hand, assessments of male gene flow during recovery of the brown bear from near extinction in Scandinavia suggest that male gene flow probably had little or no impact on the demographic recovery [36]. Lack of wide-ranging male gene flow during the short time of the recovery process resulted in low haplotype diversity and a low degree of haplotype admixture for Y-chromosomal DNA in post-bottleneck populations, suggesting that both males and females contributed to large-scale genetic connectivity in this case.

It has been hypothesized that both male and female brown bears together colonized Hokkaido from the Eurasian Continent multiple times during those periods. There may have been multiple patrilineal lineages of the brown bear in Hokkaido in the past, as with matrilineal lineages, without considering the male-biased dispersal of the brown bear. If this colonization process had occurred and male-mediated gene flow had not played a role for homogenizing patrilineal genetic variation, patrilineal phylogeographic structure in Hokkaido comprised of multiple patrilineal lineages would be expected.

To further understand the comprehensive evolutionary history of the brown bear in northeastern Asia, it is necessary to consider sex-biased migration and the role males played in forming the populations on Hokkaido and adjacent islands in terms of their contribution to genetic variation. Here we report polymorphism in Y-chromosomal DNA sequences and Y-linked microsatellites in the brown bear populations in Hokkaido, the southern Kuril Islands (Kunashiri and Etorofu), Sakhalin, and continental Eurasia (Kamchatka Peninsula, Ural Mountains, European Russia, and Tibet). We discuss the effects of sex-biased migration on the evolutionary history of the insular and continental populations, and differences in the male genetic contribution to the population composition. Finally, we assess the strength of male-mediated gene flow among geographically adjacent insular populations in northeastern Asia.

## Methods

### Samples and DNA extraction

**Fig. 1:**
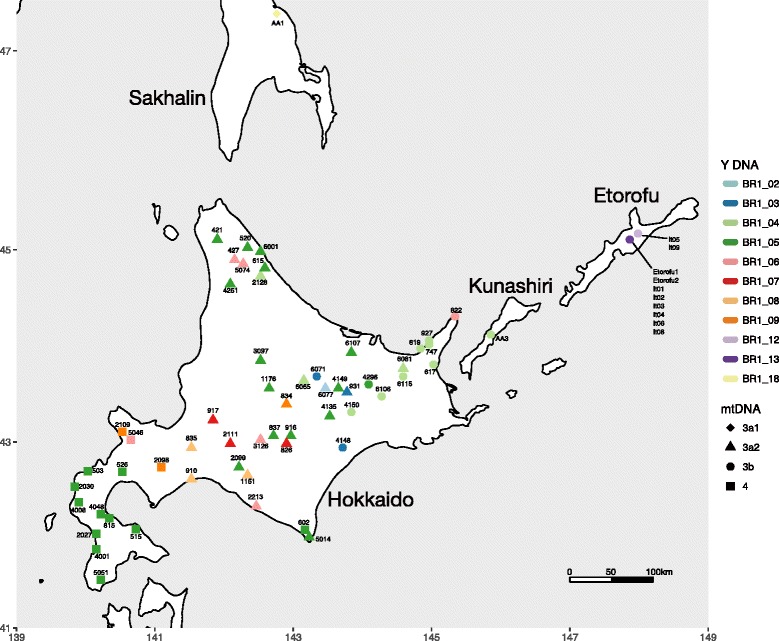
Map of Hokkaido and surrounding islands, showing the geographical distribution of brown bear Y-chromosomal compound haplotypes. Each symbol represents one bear, labeled with the sample number. Different symbols denote the maternal haplogroup (lineage) based on complete mtDNA sequences (Hirata et al. 2013). Symbol colors indicate Y-chromosomal compound haplotypes (this study)

Muscle or liver samples from 55 male brown bears collected on Hokkaido Island were obtained from the Environmental and Geological Research Department, Hokkaido Research Organization (Fig. 1). Tissue samples from males were also obtained from the following regions and sources: 10 samples from Etorofu (Iturup) Island, one from Kunashiri (Kunashir) Island, one from southern Sakhalin, and one from Novgorod (Zoological Institute, Russian Academy of Sciences, St. Petersburg); 53 from the Ural Mountains, and two from the Kamchatka Peninsula (Museum of the Institute of Plant and Animal Ecology, Russian Academy of Sciences, Ekaterinburg); and hairs of one male individual from Tibet (Kobe Municipal Oji Zoo, Japan) (Fig. 1, Additional file 1: Figures S4 and S5). The gender of samples was determined using the method of Bidon et al. [37]. Total genomic DNA was extracted with a DNeasy Tissue & Blood Kit (QIAGEN) or QIAamp DNA Micro Kit (QIAGEN), following the manufacturer’s protocols. PCR amplifications were performed in 5 μl reaction volumes, each containing 2.5 μl of 2 × Multiplex PCR Master Mix (QIAGEN), 0.5 μl of primer mixture, 0.25 μl of bovine serum albumin (BSA; 0.4 μg/μl), 0.75 μl of distilled water, and 1.0 μl of DNA extract.

### PCR amplification, sequencing, and microsatellite genotyping

Female brown bear samples were included in each round of PCR amplification as a control to confirm male specificity. No amplification was observed from female samples in any round of amplification.

The same sequencing primer sets and touchdown thermal cycling conditions as described in Bidon et al. [34] were used to amplify seven Y-linked sequence fragments (318.2C, 318.3C, 318.7C, 318.10B, 318.11C, 579.1B, and 579.3C). Touchdown PCR amplifications were conducted in 10 μl reaction volumes each containing 2.0 μl of 5 × PrimeSTAR GXL DNA Buffer (Takara), 0.8 μl of dNTP mixture (2.5 mM each dNTP; Takara), 0.2 μl of PrimeSTAR GXL DNA Polymerase (1.25 U/μl, Takara), 0.1 μl each of forward and reverse primers (25 pmol/μl), 0.2 μl of BSA (0.4 μg/μl), 5.2–6.2 μl of distilled water, and 1.0–2.0 μl of DNA extract. Touchdown thermal cycling conditions were 3 min at 95 °C; 10 cycles of 30 s at 94 °C, 25 s at 69 °C, 66 °C (decreasing by 0.5 °C per cycle), or 68 °C (decreasing by 1 °C per cycle), and 75 s at 72 °C; 25 cycles of 30 s at 94 °C, 25 s at 64 °C, 61 °C, or 58 °C, and 75 s at 72 °C; and a final extension for 10 min at 72 °C. PCR products were purified with a QIAquick Purification Kit (QIAGEN), following the manufacturer’s protocol. DNA cycle sequencing was performed with BigDye v3.1 Cycle Sequencing Kit (Applied Biosystems, ABI), using the same primers as for PCR amplification. PCR for sequencing was performed in 10 μl volumes each containing 1.75 μl of 5 × BigDye Sequencing Buffer (ABI), 0.5 μl of Ready Reaction Premix (ABI), 1.6 μl of primer (1 pmol/μl), 5.15 μl of distilled water, and 1.0 μl of DNA template. Twenty-five cycles of 10 s at 96 °C, 5 s at 50 °C, and 4 min at 60 °C were performed. Amplified DNA fragments were purified with isopropanol, and then formamide was added. Sequences were determined on an ABI 3730 DNA Analyzer, assembled and edited with phred/phrap/chromaseq [38–40], and aligned by using MUSCLE [41] in MEGA7 [42].

Nine Y-linked microsatellite alleles (Y318.1, Y318.2, Y318.4, Y318.6, Y318.9, Y369.1, Y369.4, Y69217.1, and Y15020.1) were determined by using two sets of multiplex PCRs, with the same primer sets as given in Bidon et al. [34]. Each multiplex PCR was performed in a 5 μl reaction volume containing 2.5 μl of 2 × Multiplex PCR Master Mix (QIAGEN), 1.0 μl of primer mixture, 0.25 μl of BSA (0.4 μg/μl), 0.25 μl of distilled water, and 1.0 μl of DNA extract. Touchdown thermal cycling conditions were 3 min at 95 °C; 20 cycles of 30 s at 94 °C, 25 s at 68 °C (decreasing by 0.5 °C per cycle), and 75 s at 72 °C; 15 cycles of 30 s at 94 °C, 25 s at 58 °C, and 75 s at 72 °C; and a final extension for 10 min at 72 °C. Y-linked microsatellites were determined with an ABI 3730 DNA Analyzer and the GeneScan 600 LIZ Size Standard (ABI). Microsatellite allele sizes were determined by using GeneMapper v4.1 (ABI). Nine Y-chromosome microsatellites (Y318.1, Y318.2, Y318.4, Y318.6, Y318.9, Y369.1, Y369.4, Y69217.1, and Y15020.1) were genotyped. Three of the microsatellites (Y369.4, Y69217.1, and Y15020.1) were excluded as some individuals had pseudoheterozygous genotypes, leaving six of the nine markers (Y318.1, Y318.2, Y318.4, Y318.6, Y318.9, and Y369.1); allele sizes are shown in Additional file 2: Table S1 for subsequent analysis.

### Data analyses

#### Summary statistics

Data on brown bears, polar bears, and American black bears were added to our dataset from Bidon et al. [34]. In Bidon et al. [34], all 90 individuals were sequenced for 3078 bp (3.1 kb) Y-chromosomal sequences, whereas only 44 individuals were sequenced for 5294 bp (5.3 kb) Y-chromosomal sequences. Thus we used 5.3 kb Y-chromosomal sequences for the calculation of summary statistics and network reconstruction to maximize the number of sequence length for increasing the analysis resolution. For the remaining analyses, we used 3.1 kb Y-chromosomal sequences combined with Y-linked microsatellites to maximize the number of samples to cover more geographical ranges of the brown bears. Insertions and deletions (indels) were removed from the aligned sequences. The data set comprised 5287 bp (5.3 kb data set) of Y-chromosomal sequences from 168 brown bears. Summary statistics were calculated in DnaSP version 5.10.1 [43] and Arlequin ver. 3.5.2.2 [44], including the number of haplotypes (H), frequency of the dominant haplotype (fH), number of segregating sites (S), nucleotide diversity (*π*), Watterson’s *θ*_*W*_ (per site), Tajima’s *D*, Fu and Li’s *D* and *F*, and Fu’s *F*_*S*_. Compound haplotypes were determined based on the combination of Y-linked SNPs in 3071 bp (3.1 kb data set) of Y-chromosomal DNA and six Y-linked microsatellite alleles. The genetic diversity of Y-chromosomal compound haplotypes was determined based on 214 brown bears, with the number of haplotypes (H), haplotype diversity (HD ± SD), and mean number of pairwise differences (MPD ± SD) calculated for each population. To assess the population size reductions, the modified Garza–Williamson (GW) index (*M* = *k*/*r* + 1 where *k* is the number of alleles and *r* is the range in allele size) was calculated using Arlequin ver. 3.5.2.2 [44, 45]. The GW index is small in populations that have experienced a recent bottleneck (< 0.68) and close to one in stationary populations [45].

#### Haplotype network analysis

A median-joining (MJ) network [46] of Y-chromosomal DNA compound haplotypes combined with the Y-linked SNPs in the 3.1 kb dataset and Y-linked microsatellites was reconstructed by using Network 5.0.0.0 (http://www.fluxus-engineering.com). For network calculations, more quickly evolving microsatellite loci were weighted inversely to their variance in repeat length (318.9 = 8, 318.4 = 9, 318.2 = 8, 369.1 = 2, 318.1 = 9, and 318.6 = 1), and SNP loci were weighted 10 times the greatest microsatellite weight (Y-linked SNPs = 90) as per [15]. MJ networks were constructed for the 3.1 kb and 5.3 kb data sets by using POPART [47]. The nomenclature of haplotypes based on the 5.3 kb and 3.1 kb data sets corresponds to that of Bidon et al. [34]. Names of Y-chromosomal DNA compound haplotypes refer first to the 3.1 kb of Y-chromosomal DNA and then to the genotype from six microsatellite loci. The three sub-populations on Hokkaido Island (central, eastern, and southern) correspond to mtDNA clades 3a2, 3b, and 4, respectively, from Matsuhashi et al. [28] and Hirata et al. [25].

#### Population differentiation analysis

Pairwise population differentiation values (*R*_ST_) for Y-chromosomal polymorphisms [48] were calculated with 1000 permutations by using Arlequin ver 3.5.2.2 [44]. Hierarchical analysis of molecular variance (AMOVA) was implemented in Arlequin ver 3.5.2.2. Geographical partitioning of Y-chromosomal polymorphisms was also tested by AMOVA, with partitions defined as the North American, Eurasian, Hokkaido, and Etorofu groups. Based on a preliminary AMOVA analysis, the Etorofu population was included in the Eurasian group for further analyses. The three populations in the Hokkaido group (central, eastern, and southern) were defined by mtDNA lineages as described above. Brown bear populations represented by only one individual (Tibet, Sakhalin, and Kunashiri) were excluded from the AMOVA analysis.

#### TMRCA estimations by Bayesian analysis

Times to the most recent common ancestors (TMRCAs) and the times of population splitting were estimated by using the Bayesian-based coalescent approach implemented in the software BATWING [49]. Time estimation for Y-chromosomal lineages was implemented by including Y-linked SNPs treated as unique event polymorphism sites together with Y-linked microsatellite genotypes. Coalescence times were estimated for all brown bears, the Hokkaido and Etorofu brown bears, only Hokkaido brown bears, and only Etorofu brown bears. Single population model was specified for the TMRCA estimation and multiple population models were specified for the splitting time calculation. None of the neutrality tests was significantly different from the expectation under neutrality; thus, all of the analysis was implemented under the assumption of constant population size. Priors applied were a mean mutation rate of 6.9 × 10^−4^ mutations per locus per generation determined for human Y-chromosomal microsatellite DNA, with the gamma distribution [50] according to the method of Wei et al. [8], and a mean effective population size of 10,000, with the gamma distribution as per [15]. For each BATWING run, one million MCMC cycles were performed, with the first 10% of each run discarded as burn-in. All runs achieved effective sample sizes >200 for all parameters and the convergence of replicate MCMC runs were confirmed in Tracer v1.6.0 [51]. A generation time of 10 years was assumed for the brown bear [52] to allow conversion of generations into years. In the BATWING analyses, all brown bear individuals were included in the data set. Geographical population subdivisions were defined as Eurasia, North America, Hokkaido (treated as one population), and Etorofu. One individual from Kunashiri was included in the Hokkaido population and one individual from Sakhalin was included in the Eurasian population.

## Results

### Polymorphism and genetic diversity

No Y-chromosomal haplotypes were shared among brown bears, polar bears (*U. maritimus*), and American black bears (*U. americanus*), and each of these species was reciprocally monophyletic for both the 5.3 kb and 3.1 kb data sets (Additional file 1: Figures S1 and S2). In the 3.1 kb data set, including data from Bidon et al. [34], six haplotypes (BR1, BR2, BR3, BR4, BR5, and BR6) were found among 214 brown bears (Additional file 1: Figure S1). Novel haplotype BR6 was detected in four individuals from the Ural Mountains. Except for 11 individuals having minor haplotypes (BR2, BR3, BR4, BR5, and BR6), 207 individuals shared haplotype BR1, including all individuals from Hokkaido.

In the 5.3 kb data set, including data from Bidon et al. [34], nine haplotypes (BR1.1, BR1.2, BR1.4, BR1.5, BR2, BR3, BR5, BR6.1, and BR6.2) discriminated by nine segregating sites were found among 168 brown bears (Additional file 1: Figure S2). We identified four novel haplotypes (BR1.4, BR1.5, BR6.1, and BR6.2). The maximum number of nucleotide differences between haplotypes was five. BR1.4 was the most common haplotype, found in all brown bears from Hokkaido and in 98 individuals (58% of total) from Hokkaido, Kunashiri, Etorofu, Sakhalin, Tibet, the Ural Mountains, Kamchatka, and European Russia. Three haplotypes (BR1.5, BR6.1, and BR6.2) were detected only in brown bears from Ural Mountains.

Nucleotide diversity (*π*) and Watterson’s *θ*_*W*_ per site for all brown bears were (1.5 ± 0.1) × 10^−4^ and (3.0 ± 1.2) × 10^−4^, respectively. None of the four neutrality indices for the 5.3 kb data set was significantly different from the expectation under neutrality, nor did paternal brown bears experience a recent population expansion or contraction: Tajima’s *D* = −1.19 (*P* > 0.10), Fu and Li’s *D* = −1.95 (*P* > 0.05), Fu and Li’s *F* = −2 (*P* > 0.05), Fu’s *Fs* = −3.548 (*P* > 0.05).

In all, 80 compound haplotypes, defined by SNPs in the 3.1 kb data set and the six microsatellites alleles, were found among 214 individuals (Additional file 2: Table S1), and 39 haplotypes were novel. Among the brown bear populations in western Asia, including the Ural Mountains, 32 haplotypes were detected, and both the haplotype diversity (HD = 0.96 ± 0.01) and mean number of pairwise differences (MPD = 3.59 ± 1.85) were highest among the various regions (Table 1). A total of eight haplotypes were found among 55 individuals on Hokkaido. The MPD for Hokkaido was nearly one-third that of Western Asia, and was the lowest among all populations, except for Canada. Both genetic diversity indices for Hokkaido were lower (HD = 0.73 and MPD = 1.23) than those for the North American and Eurasian continental populations, except for Canada. On Hokkaido, both HD and MPD were highest in central Hokkaido and lowest in southern Hokkaido (Table 1). All eight haplotypes detected on Hokkaido were represented in the central Hokkaido population. On Etorofu Island, only two haplotypes different by one microsatellite mutational step were found among 10 individuals, and the MPD was the lowest among all brown bear populations where multiple individuals were investigated. The GW index was calculated for each geographic population, and ranged from 0.75 (eastern Hokkaido) to 1.00 (Etorofu) (Table 1). The GW index of all geographic populations was greater than critical value of 0.68 for the indication of a recent population bottleneck and did not deviate from putatively stable populations [45].

**Table 1:** Paternal genetic diversity in various geographical populations of brown bears, based on 3.1-kb Y-chromosomal nucleotide sequences and six Y-linked microsatellite loci

Population	n	H	HD ± SD	MPD ± SD	GW ± SD
All brown bears	214	79	0.97 ± 0.01	4.08 ± 2.04	–
Northwest America (NW-A)^a^	10	6	0.84 ± 0.10	2.31 ± 1.38	0.92 ± 0.20
ABC Islands (ABC)^a^	11	5	0.82 ± 0.08	1.82 ± 1.13	0.93 ± 0.12
Canada (CAN)^a^	8	2	0.25 ± 0.18	0.75 ± 0.61	0.89 ± 0.19
Central Europe (C-EU)^a^	14	8	0.89 ± 0.06	2.68 ± 1.52	0.93 ± 0.15
Northern Europe (N-EU)^a^	11	5	0.78 ± 0.11	2.62 ± 1.51	0.94 ± 0.14
Western Asia (W-AS)	61	32	0.96 ± 0.01	3.59 ± 1.85	0.97 ± 0.08
East Asia (E-AS)	31	14	0.86 ± 0.05	2.60 ± 1.43	0.94 ± 0.16
Sakhalin (SH)	1	1	1.00	0.00	–
Tibet (TB)	1	1	1.00	0.00	–
Etorofu (ET)	10	2	0.36 ± 0.16	0.36 ± 0.38	1.00 ± 0.00
Kunashiri (KN)	1	1	1.00	0.00	–
Hokkaido (HK)	55	8	0.73 ± 0.05	1.23 ± 0.79	0.83 ± 0.14
Central Hokkaido (C-HK)	30	8	0.76 ± 0.07	1.26 ± 0.82	0.83 ± 0.14
Eastern Hokkaido (E-HK)	11	4	0.60 ± 0.15	0.85 ± 0.65	0.75 ± 0.35
Southern Hokkaido (S-HK)	14	3	0.38 ± 0.15	0.41 ± 0.40	0.83 ± 0.24

^a^Citations from Bidon et al. [34]

*n* sample size, *H* number of haplotypes, *HD* haplotype diversity, *MPD* mean number of pairwise differences within population, *GW* modified Garza–Williamson index, *SD* standard deviation

### Haplotype networks

**Fig. 2:**
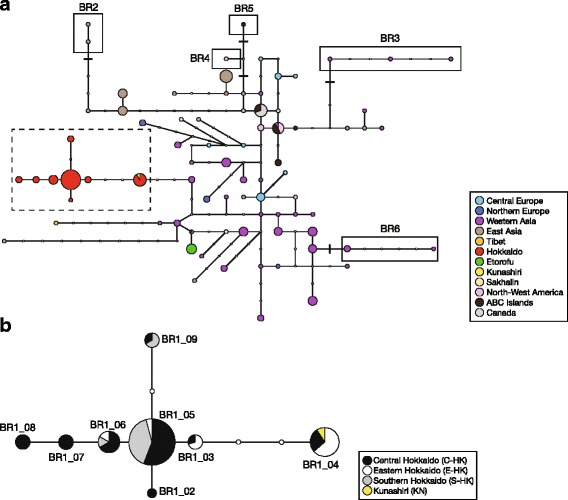
**a** Median-joining haplotype network for brown bears, based on Y-chromosomal compound haplotypes combined with Y-linked SNPs from a 3.1 kb data set and Y-linked microsatellites. Haplotypes enclosed by a dashed line are from Hokkaido (including one Kunashiri brown bear). Haplotypes enclosed by solid lines denote the same haplotypes (BR2–BR6) as those distinguished by only the 3.1 kb Y-linked data set. The remaining haplotypes have the same haplotype (BR1) distinguished by only the 3.1 kb data set. Each color represents a different brown bear population. Each black bar crossing a network line denotes a single mutational step revealed by a single nucleotide polymorphism. Small, open circles indicate intermediate haplotypes not actually observed; each line connecting two haplotype circles represents single microsatellite mutational steps. The size of each colored haplotype circle is proportional to the number of individuals having that haplotype. **b** MJ network for Hokkaido brown bears (including one from Kunashiri Island), based on the same data as above. Symbols and conventions are as for (**a**)

Among all brown bear populations, only the Y-chromosomal haplotypes from Hokkaido showed clear geographic clustering in the haplotype network (Fig. 2a and Additional file 1: Figure S3), sharing no haplotypes with continental Eurasian and North American populations; other populations showed no clear geographic structure. Y-chromosomal haplotypes were shared among the three Hokkaido populations, and their geographical distribution was not congruent with the population delineation by mtDNA (Figs 1 and 2b). Three male individuals (ID 602, 6071, and 6081 representing mtDNA clades 4, 3b, and 3a2, respectively) had outlying maternal haplotypes that were found within populations defined by other maternal haplotypes (mtDNA clades 3a2, 3a2, and 3b, respectively) (Fig. 1). These individuals had paternal haplotypes that in some cases were detected in presumed natal populations and in other cases dispersed across maternal population boundaries.

On Hokkaido Island, the paternal haplotype distribution did not show any allopatric separation, although a somewhat biased haplotype distribution was observed (Figs. 1 and 2b). All of the paternal haplotypes detected on Hokkaido were represented in the central population, which thus showed higher haplotype diversity than the eastern and southern populations. Haplotype BR1_05 had the highest frequency on Hokkaido and was found in all three populations (Fig. 2b). Haplotype BR1_06, which was one microsatellite mutational step away from major haplotype BR1_05, was also detected in all three populations. Minor haplotypes BR1_02, BR1_07, and BR1_08, differing from BR1_06 by 1–3 microsatellite mutational steps, were found only in the central population.

Among populations in the southern Kuril Islands close to Hokkaido, one brown bear on Kunashiri Island shared haplotype BR1_04 with Hokkaido bears (Figs. 1 and 2b), and the eastern Hokkaido population had the highest frequency of this haplotype. In contrast, the two haplotypes detected on Etorofu Island were more closely related to haplotypes in continental Eurasia than to those on Hokkaido (Figs. 1 and 2a). Etorofu shared haplotype BR1_12 with the Ural Mountains population, while BR1_13 was restricted to Etorofu Island. On Sakhalin, haplotype BR1_18 was closely related to haplotypes in continental Eurasia. One Tibetan brown bear had a distinct haplotype (BR1_10) that differed from BR1_11 from the Ural Mountains by six microsatellite mutational steps. Populations in the Ural Mountains and Kamchatka Peninsula had highly variable haplotypes compared with the other populations, but neither population exhibited a clear relationship between genetic relatedness and geographical location.

### Differentiation among populations

Hierarchical analyses by AMOVA were implemented for various geographical partitions of brown bear populations and groups (Table 2, Additional file 2: Tables S2, S3, and S4). Inclusion of the Etorofu population in the Eurasian Continent group resulted in higher among-group variance (44.43%, *P* < 0.001) than when it was included in the North American (41.35%, *P* < 0.001) or Hokkaido (23.51%, *P* = 0.067) groups (Table 2). The Etorofu population is thus more closely related to the continental Eurasian population than with the North American or Hokkaido populations. When the continents (Eurasia and North America) and Hokkaido were partitioned into different groups, the proportion of among-group variance was highest (54.68%, *P* < 0.001), and the differentiation was more pronounced. The AMOVA results indicated that the Hokkaido group was highly differentiated from the Eurasian Continent (including Etorofu) and North American groups. There was no genetic connectivity between the Hokkaido group and the continental groups. In addition, there was no substantial population differentiation among the central, eastern, and southern Hokkaido populations.

**Table 2:** Analysis of molecular variance (AMOVA) for Y-chromosomal polymorphisms in various geographical partitions

Geographical partitions	Sum of squares	Variance components	Percentage of variance (%)
(EU + ET), NA, and HK (N_Groups_ = 3)			
Among groups	5290.74	38.71*	44.43
Among population, within groups	2729.17	17.89*	20.54
Within populations	6104.21	30.52*	35.03
EU, (NA + ET), and HK (N_Groups_ = 3)			
Among groups	5212.04	34.79*	41.35
Among population, within groups	2807.87	18.81*	22.36
Within populations	6104.21	30.52*	36.28
EU, NA, and (HK + ET) (N_Groups_ = 3)			
Among groups	3597.58	18.68	23.51
Among population, within groups	4422.33	30.24*	38.06
Within populations	6104.21	30.52*	38.42
[(EU + ET), NA], HK (N_Groups_ = 2)			
Among groups	5122.78	57.32*	54.68
Among population, within groups	2897.13	16.99*	16.21
Within populations	6104.21	30.52*	29.12
Within (HK + ET) (N_Groups_ = 1)			
Among populations	2256.75	49.72*	82.27
Within populations	653.90	10.72	17.73
Within HK (N_Groups_ = 1)			
Among populations	235.27	6.40*	33.95
Within populations	647.50	12.45	66.05

*P < 0.001

*EU* Eurasian Continent (Central Europe, Northern Europe, Western Asia, Eastern Asia), *NA* North American Continent (Northwest America, ABC Islands, Canada), *HK* Hokkaido (Central Hokkaido, Eastern Hokkaido, Southern Hokkaido), *ET* Etorofu Island

Although Etorofu Island is geographically close to Kunashiri and Hokkaido islands, it was significantly differentiated from the central, eastern, and southern Hokkaido populations (*R*_ST_ = 0.92, 0.89, and 0.98, respectively), but least differentiated from the East Asia population (*R*_ST_ = 0.18) (Table 3). The three Hokkaido populations were significantly differentiated from all three North American and all five Eurasian Continental populations. Among the Hokkaido populations, however, the eastern population was significantly differentiated from the central and southern populations (*R*_ST_ = 0.42 and 0.58, respectively), whereas the latter two were not differentiated from one another (*R*_ST_ = 0.04).

**Table 3:** Pairwise population differentiations (*R*_*ST*_) for Y-chromosomal DNA markers among brown bear populations

	NW-A	ABC	CAN	C-EU	N-EU	W-AS	E-AS	ET	C-HK	E-HK	S-HK
NW-A											
ABC	−0.03										
CAN	−0.08	0.05									
C-EU	−0.004	0.11	−0.06								
N-EU	0.37*	0.41*	0.36*	0.26*							
W-AS	0.169*	0.25*	0.17*	0.14*	0.22*						
E-AS	0.19*	0.24*	0.18*	0.23*	0.43*	0.45*					
ET	0.73*	0.79*	0.86*	0.59*	0.85*	0.48*	0.18*				
C-HK	0.79*	0.77*	0.81*	0.80*	0.77*	0.72*	0.68*	0.92*			
E-HK	0.63*	0.59*	0.72*	0.64*	0.67*	0.53*	0.48*	0.89*	0.42*		
S-HK	0.89*	0.87*	0.93*	0.84*	0.86*	0.74*	0.65*	0.98*	0.04	0.58*	

*P < 0.05

Excluding the Etorofu population from the Eurasian Continental group, *R*_ST_ values between the North American and Eurasian Continental populations were lower than those between the North American and Hokkaido populations, or between the Eurasian Continental and Hokkaido populations.

### Bayesian estimates of divergence times

BATWING was used to estimate the time to the most recent common ancestor (TMRCA) and effective population size (*N*_*e*_) for paternal lineages based on Y-linked SNPs in the 3.1 kb data set and six Y-linked microsatellites (Table 4). The mean TMRCA for all males was 472.7 kyBP (thousands of years before present). That for the Hokkaido and Etorofu populations was 127.8 kyBP, slightly older than the splitting time between the Hokkaido brown bears and the other populations. The mean TMRCA of the Hokkaido population was 55.3 kyBP with an effective population size of 1723 (569–4380). The splitting time between the Etorofu brown bears and the other populations was 36.9 kyBP, and the TMRCA of the Etorofu brown bears was 4.4 kyBP with an effective population size of 295 (31–1125), which was less than one-fifth the size of the Hokkaido population.

**Table 4:** Time to the most recent common ancestor (TMRCA) in thousands of years before present (kyBP), estimated from Y-chromosomal DNA markers by BATWING analysis, scaled using effective population size (N_e_)

	TMRCA (mean)	Splitting time (mean)	95% Credible interval	N_e_ (mean)	95% Credible interval
All brown bears	472.7	–	186.8–1048.8	31,520	16,463–55,764
HK – (NA + EU + ET)	–	124.6	16.5–645.6	–	–
ET – (NA + EU + HK)	–	36.9	1.0–277.6	–	–
HK + ET	127.8	–	40.3–332.1	2457	865–6051
HK	55.3	–	15.7–153.9	1723	569–4380
ET	4.4	–	0.5–15.8	295	31–1125

*EU* Eurasian Continent (Central Europe, Northern Europe, Western Asia, Eastern Asia, Sakhalin), *NA* North American Continent (Northwest America, ABC Islands, Canada), *HK* Hokkaido (Central Hokkaido, Eastern Hokkaido, Southern Hokkaido), *ET* Etorofu Island

## Discussion

The paternal phylogeographic structure of brown bears across northeastern Asia reconstructed by using Y-chromosomal DNA polymorphisms contrasted with the maternal phylogeographic structure not only in continental Eurasia and North America, but also around Hokkaido and adjacent islands. Sex-biased dispersal by the brown bear could have markedly affected the insular brown bear populations, and differentially affected the evolutionary history in the insular and continental populations. The hypothesis that both male and female brown bears colonized from the Eurasian Continent to Hokkaido multiple times together was supported; however, male-mediated gene flow played a role for homogenizing patrilineal genetic variation and resulted in the geographically indistinct paternal phylogeographic structure of brown bears in Hokkaido.

### Paternal phylogeography on Hokkaido Island

Based on paternal DNA, Hokkaido brown bears were highly differentiated from populations in continental Eurasia and North America, indicating a lack of genetic connectivity with the continental populations (Fig. 2a; Tables 2 and 3). The paternal lineage of the Hokkaido brown bears split off from the continental brown bears an estimated 124.6 kyBP (16.5–645.6 kyBP) (Table 4). In contrast, there was only weak phylogeographic structuring of paternal haplotypes throughout continental Eurasia and North America, probably resulting from male-mediated gene flow across continents [34]. The paternal lineages on Hokkaido and the continents may have evolved independently, resulting in different evolutionary histories.

In North America, male-mediated gene flow connects populations on the Alaskan ABC islands with those on the North American mainland and plays an important role in maintaining high genetic variation in the insular populations [34]. In contrast, there was no direct evidence of male-mediated gene flow across the sea straits between Hokkaido Island and continental Eurasia. Male-mediated gene flow from the continent appears to have played little role in the patrilineal genetic variation in the Hokkaido population after the separation of Hokkaido Island by the opening of sea straits following the last glacial maximum (LGM). Instead, substantial genetic drift on Hokkaido Island appears to have contributed to paternal genetic diversity and differentiation between the Hokkaido and continental populations. The influence of male-mediated gene flow on the genetic diversity of island populations thus appears to differ from region to region.

While a network of Y-chromosomal haplotypes identified the Hokkaido population as distinct from populations in other regions, there was little geographic structuring within Hokkaido (Figs. 1 and 2). This is incongruent with the results of mtDNA analyses showing three distinct maternal lineages allopatrically distributed on Hokkaido [25, 28]. This incongruence suggests that strongly male-biased, long distance dispersal played a significant role in the genetic makeup of the brown bear population. The paternal genetic diversity was much lower on Hokkaido than in the continental populations in North America and Eurasia, indicating that male-biased dispersal was a stronger factor in homogenizing genetic diversity on the restricted small island than across continents.

The paternal lineage of Hokkaido brown bears showed a much more recent coalescence than the maternal lineages. The TMRCA for the paternal lineage was estimated at 55.3 kyBP (15.7–153.9 kyBP) (Table 4). In contrast, Hirata et al. [25] estimated the TMRCA for mtDNA lineages in the Hokkaido population to be 268 kyBP (109–457 kyBP), nearly five times that of the paternal coalescence. The central, eastern, and southern maternal lineages coalesced approximately 27 kyBP (10–49 kyBP), 42 kyBP (14–80 kyBP), and 36 kyBP (12–67 kyBP), respectively, and were more similar to the coalescence time for paternal lineage. Polygyny among brown bears may have led the small male effective population size and caused the much more recent coalescence of paternal lineages. In addition, the difference in TMRCA between the maternal and paternal lineages can be explained by the pronounced sexual bias in brown bear dispersal behavior. Since the brown bear population on Hokkaido formed through the immigration of three different lineages from continental Eurasia in different periods [25], one might expect also to find highly diverged paternal haplotypes descended from the three past pulses of immigration into Hokkaido. Contrary to this expectation, the Y-chromosomal DNA haplotypes detected on Hokkaido were more recently diverged, differing by only a few mutational steps in microsatellites (Figs. 1 and 2; Additional file 2: Table S1). In the 3.1 kb and 5.3 kb data sets, all individuals from Hokkaido shared haplotypes BR1 and BR1.4, respectively. No relatively old haplotypes discriminated by more slowly evolving SNPs were found, although these might have been expected considering multiple past pulses of immigration from continental Eurasia. Highly diverged patrilineal lineages previously immigrated into Hokkaido were overridden by more recent immigrant lineage and relatively old haplotypes must have become extinct on Hokkaido. Thus, all paternal haplotypes shared by the Hokkaido brown bears are specific to Hokkaido Island.

We detected one major haplotype (BR1_05) throughout Hokkaido Island (Figs. 1 and 2b; Additional file 2: Table S1). As mentioned in Results, three male individuals (ID 602, 6071, and 6081) had maternal haplotypes that were inconsistent with population region where they were collected, as defined by mtDNA (Fig. 1). These males were probably individuals that had dispersed from their natal area during a single generation. Other studies [53, 54] using a combination of mtDNA and autosomal microsatellite markers have also demonstrated dispersal of extant males as well as male-mediated gene flow between natal areas defined by mtDNA lineages (the southern Akan-Shiranuka region, central and eastern Hokkaido). These signs of male-mediated gene flow among populations reiterate that the distinct maternal phylogeographic structure on Hokkaido Island has been maintained by strong female philopatric behavior, despite fairly common male immigration between maternal populations.

Although paternal population structuring was weak, differential and biased pairwise population differentiation was evident among the three Hokkaido populations (Table 3), suggesting a tendency toward biased dispersal and mixing of males from past to present. There was no paternal population differentiation between the central and southern Hokkaido populations, which indicates that frequent movement of males between these populations caused interchange of paternal haplotypes. On the other hand, significant population differentiation between eastern Hokkaido and each of the other two regions suggests some restriction of brown bear migration between these populations. Morphometric data also suggest some differentiation of males between geographical populations defined by mtDNA markers. Although female cranial characters were markedly differentiated among the populations, males were similar between the central and southern Hokkaido populations, whereas males in eastern Hokkaido were distinct [55]. The Shiretoko Peninsula in eastern Hokkaido has highest density of the brown bears on Hokkaido Island. The high bear density may have induced these males to exhibit more sedentary behavior than in other areas and restricted male-mediated gene flow into this area.

### Paternal phylogeography in the southern Kuril Islands

The maternal lineage in the southern Kuril Islands (Kunashiri and Etorofu) apparently originated from eastern Hokkaido [25]. Contrary to expectations based on mtDNA analyses that the paternal lineage on Etorofu would have the same demographic history as that on Kunashiri, we detected lineage differentiation between Hokkaido/Kunashiri and Etorofu. The two haplotypes detected on Etorofu were more closely related to haplotypes from continental Eurasia than to those from Hokkaido, even though a large sample (*n* = 55) from across Hokkaido was genotyped (Fig. 2a; Tables 2 and 3). The TMRCA for the Hokkaido + Etorofu male populations (127.8 kyBP) was older than for the matrilineal lineage and was roughly contemporaneous with the split of the Hokkaido lineage from the continental lineages (124.6 kyBP) (Table 4). In the maternal lineage, brown bears from both southern Kuril Islands were estimated to have diverged from the eastern Hokkaido lineage less than 42 kyBP (14–80 kyBP) [25]. The estimated male effective population size on Etorofu Island was much smaller than that on Hokkaido Island. A small number of individuals may have contributed to the makeup of the population on Etorofu.

Y-chromosomal DNA was genotyped for ten individuals from Etorofu, of which four had been included in an analysis of complete mtDNA sequences [25]; three of the four individuals were identical in mtDNA haplotype, excluding the fast-evolving variable-number tandem repeats in the control region. Most brown bears on Etorofu likely belong to the same maternal lineage. Only two Y-chromosomal DNA haplotypes differing by one microsatellite mutational step were found among 10 individuals from Etorofu Island, giving the lowest genetic diversity of among populations (Fig. 2a; Table 1). Etorofu Island brown bears also likely comprise the paternally related lineage. Geologically, Kunashiri Island was connected to Hokkaido by a land bridge 8–110 kyBP, whereas Etorofu Island remained separate during that period [56]. The brown bear population on Etorofu may have been maintained by inbreeding for a long time, with a founder event and subsequent genetic drift leading to the low diversity we observed. In contrast to the maternal lineages, the present-day paternal lineage on Etorofu possibly originated by the dispersal of male individuals from continental Eurasia. Thus, the different dispersal behaviors of male and female brown bears appear to have contributed to the makeup of the Etorofu population, in which maternal and paternal lineages had different origins. Furthermore, there appears to have been little recent male-mediated gene flow between Hokkaido/Kunashiri and Etorofu.

### Paternal phylogeography on the Eurasian continent

Brown bear populations in the Ural Mountains and Kamchatka Peninsula had higher haplotype variation than other populations, but neither population showed a clear relationship between genetic relatedness and geographical location. Western Asia, including the Ural Mountains, had the highest index values for paternal genetic diversity in our study (Table 1), whereas only one mtDNA lineage (clade 3a1) has been detected in the same region, with relatively low maternal genetic diversity [25, 26, 29–31]. High paternal genetic variation within populations in this region, compared to low genetic differentiation among the populations, supports the conclusion that male-mediated gene flow contributed highly to the brown bear population history in continental Eurasia, especially around the Ural Mountains.

## Conclusions

Brown bears on Hokkaido and the adjacent southern Kuril Islands experienced different maternal and paternal evolutionary histories, demonstrating that the phylogeography in this region is considerably more complicated than would be expected from mtDNA studies alone. The weak spatial structuring of paternal lineages detected on Hokkaido appears to have resulted from extensive, continual gene flow via male dispersal among natal populations after the last population immigrated into Hokkaido from eastern Siberia via a land bridge during the last glacial period. The paternal genetic structure did not show distinct allopatric lineages, as have been observed for maternal (mtDNA) markers. While there were indications of heterogeneous male-mediated gene flow among populations on Hokkaido, it is unclear how much this influenced the connectivity and the maintenance of the local populations. Our results indicate that sex-biased dispersal has played a significant role in the evolutionary history of the brown bear in continental populations and in peripheral insular populations such as on Hokkaido, the southern Kuril Islands, and Sakhalin. Biparentally inherited autosomal DNA and whole-genomic data could further clarify the detailed demographic history and local adaptation of Asian brown bears.

## Additional files

Additional file 1: Figure S1. Median-joining haplotype network for brown, polar, and American black bears, based on the 3.1 kb Y-linked data set. **Figure S2.** Median-joining haplotype network for brown, polar, and American black bears, based on the 5.3 kb Y-linked data set. **Figure S3.** Median-joining haplotype network for brown bears, based on Y-chromosomal compound haplotypes combined with Y-linked SNPs from a 3.1 kb data set and Y-linked microsatellites. Haplotypes enclosed by a dashed line are from Hokkaido (including one Kunashiri brown bear). **Figure S4.** Map of Eurasia showing the geographical distribution of brown bear Y-chromosomal compound haplotypes. Each symbol represents one individual. **Figure S5.** Enlargement of the larger boxed area in Fig. S4, showing the geographical distribution of brown bear Y-chromosomal compound haplotypes around the Ural Mountains. (ZIP 4.95 mb)

Additional file 2:Table S1. List of Y-chromosomal DNA compound haplotypes (YDNA haplotype), Y-chromosomal haplotypes based on only the 3.1 kb data set (YSNP_haplotype), Y-linked SNPs in the 3.1 kb data set, fragment sizes of six Y-linked microsatellites markers, and the number of individuals from each geographical regions having each Y-chromosomal DNA compound haplotypes. **Table S2.** Analysis of molecular variance (AMOVA) for brown bear Y-chromosomal polymorphisms in various geographical partitions. **Table S3.** Analysis of molecular variance (AMOVA) for brown bear Y-chromosomal polymorphisms in various geographical partitions. **Table S4.** Analysis of molecular variance (AMOVA) for brown bear Y-chromosomal polymorphisms in various geographical partitions. (ZIP 63 kb)
