# Supplementary material for: Paternal phylogeographic structure of the brown bear (Ursus arctos) in northeastern Asia and the effect of male-mediated gene flow to insular populations
Source: Zoological Lett. 2017 Nov 30;3:21. doi: 10.1186/s40851-017-0084-5 (PMC5707830; doi:10.1186/s40851-017-0084-5)
Supplement: Supplementary file 1 — Median-joining haplotype network for brown, polar, and American black bears, based on the 3.1 kb Y-linked data set. Figure S2. Median-joining haplotype network for brown, polar, and American black bears, based on the 5.3 kb Y-linked data set. Figure S3. Median-joining haplotype network for brown bears, based on Y-chromosomal compound haplotypes combined with Y-linked SNPs from a 3.1 kb data set and Y-linked microsatellites. Haplotypes enclosed by a dashed line are from Hokkaido (including one Kunashiri brown bear). Figure S4. Map of Eurasia showing the geographical distribution of brown bear Y-chromosomal compound haplotypes. Each symbol represents one individual. Figure S5. Enlargement of the larger boxed area in Fig. S4, showing the geographical distribution of brown bear Y-chromosomal compound haplotypes around the Ural Mountains. (ZIP 4.95 mb) [file 40851_2017_84_MOESM1_ESM.zip › 171121_Hirata_ZL_additionalfile1.pdf]

Additional file 1:

Fig. S1 Median-joining haplotype network for brown, polar, and American black bears, based on the 3.1 kb Y-linked data set. Colors indicate the bear species. Small, filled circles indicate intermediate haplotypes not actually observed; each line connecting two haplotype circles represents a single mutational step revealed by a single nucleotide polymorphism. The size of each colored haplotype circle is proportional to the number of individuals having that haplotype. Asterisks indicate novel haplotypes detected in the present study. Haplotype nomenclature corresponds to that of Bidon et al. (2014).

Fig. S2 Median-joining haplotype network for brown, polar, and American black bears, based on the 5.3 kb Y-linked data set. Colors indicate the bear species. Small, filled circles indicate intermediate haplotypes not actually observed; each line connecting two haplotype circles represents a single mutational step revealed by a single nucleotide polymorphism. The size of each colored haplotype circle is proportional to the number of individuals having that haplotype. Asterisks indicate novel haplotypes detected in the present study. Haplotype nomenclature corresponds to that of Bidon et al. (2014).

Fig. S3 Median-joining haplotype network for brown bears, based on Y-chromosomal compound haplotypes combined with Y-linked SNPs from a 3.1 kb data set and Y-linked microsatellites. Haplotypes enclosed by a dashed line are from Hokkaido (including one Kunashiri brown bear). Haplotypes enclosed by solid lines denote the same haplotypes (BR2–BR6) as those distinguished by only the 3.1 kb Y-linked data set. The

remaining haplotypes have the same haplotype (BR1) distinguished by only the 3.1 kb data set. Each color represents a different brown bear population. Each black bar crossing a network line denotes a single mutational step revealed by a single nucleotide polymorphism. Small, open circles indicate intermediate haplotypes not actually observed; each line connecting two haplotype circles represents single microsatellite mutational steps. The size of each colored haplotype circle is proportional to the number of individuals having that haplotype. This figure is identical to Fig. 1a, except that all haplotype names have been added.

Fig. S4 Map of Eurasia showing the geographical distribution of brown bear Y-chromosomal compound haplotypes. Each symbol represents one individual. Different symbols denote the maternal haplogroup (lineage) based on complete mtDNA sequences (Hirata et al. 2013). Symbol colors indicate Y-chromosomal compound haplotypes (this study).

Fig. S5 Enlargement of the larger boxed area in fig. S4, showing the geographical distribution of brown bear Y-chromosomal compound haplotypes around the Ural Mountains. Each symbol represents one individual. All individuals belong to maternal haplogroup (lineage) 3a1, based on complete mtDNA sequences (Hirata et al. 2013). Symbol colors indicate Y-chromosomal compound haplotypes (this study).
