# Supplementary figures and images for: Paternal phylogeographic structure of the brown bear (Ursus arctos) in northeastern Asia and the effect of male-mediated gene flow to insular populations
Source: Zoological Lett. 2017 Nov 30;3:21. doi: 10.1186/s40851-017-0084-5 (PMC5707830; doi:10.1186/s40851-017-0084-5)

Fig. S4

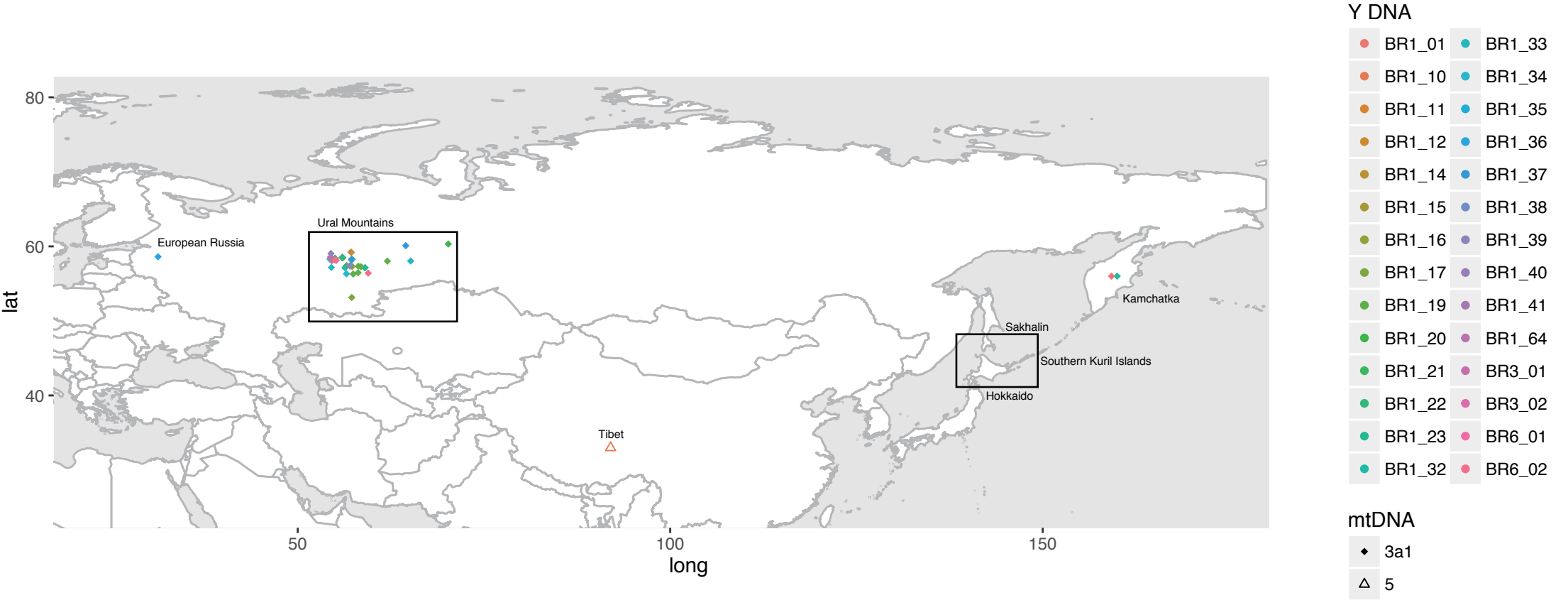

Supplement: Supplementary file 1 — Median-joining haplotype network for brown, polar, and American black bears, based on the 3.1 kb Y-linked data set. Figure S2. Median-joining haplotype network for brown, polar, and American black bears, based on the 5.3 kb Y-linked data set. Figure S3. Median-joining haplotype network for brown bears, based on Y-chromosomal compound haplotypes combined with Y-linked SNPs from a 3.1 kb data set and Y-linked microsatellites. Haplotypes enclosed by a dashed line are from Hokkaido (including one Kunashiri brown bear). Figure S4. Map of Eurasia showing the geographical distribution of brown bear Y-chromosomal compound haplotypes. Each symbol represents one individual. Figure S5. Enlargement of the larger boxed area in Fig. S4, showing the geographical distribution of brown bear Y-chromosomal compound haplotypes around the Ural Mountains. (ZIP 4.95 mb) [file 40851_2017_84_MOESM1_ESM.zip › 170811 FigS4.pdf]

Fig. S3

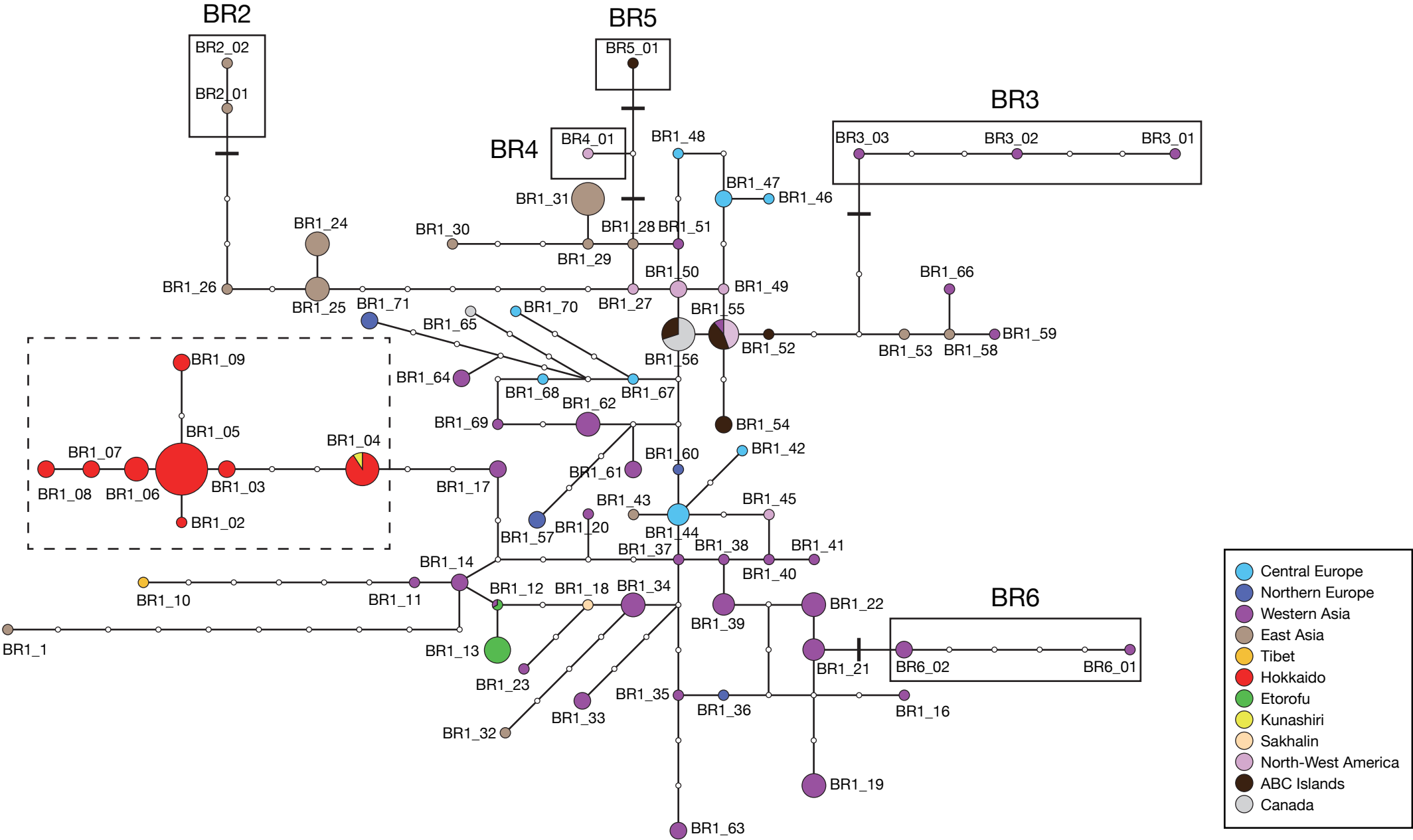

Supplement: Supplementary file 1 — Median-joining haplotype network for brown, polar, and American black bears, based on the 3.1 kb Y-linked data set. Figure S2. Median-joining haplotype network for brown, polar, and American black bears, based on the 5.3 kb Y-linked data set. Figure S3. Median-joining haplotype network for brown bears, based on Y-chromosomal compound haplotypes combined with Y-linked SNPs from a 3.1 kb data set and Y-linked microsatellites. Haplotypes enclosed by a dashed line are from Hokkaido (including one Kunashiri brown bear). Figure S4. Map of Eurasia showing the geographical distribution of brown bear Y-chromosomal compound haplotypes. Each symbol represents one individual. Figure S5. Enlargement of the larger boxed area in Fig. S4, showing the geographical distribution of brown bear Y-chromosomal compound haplotypes around the Ural Mountains. (ZIP 4.95 mb) [file 40851_2017_84_MOESM1_ESM.zip › 170811 FigS3.pdf]

Fig. S2

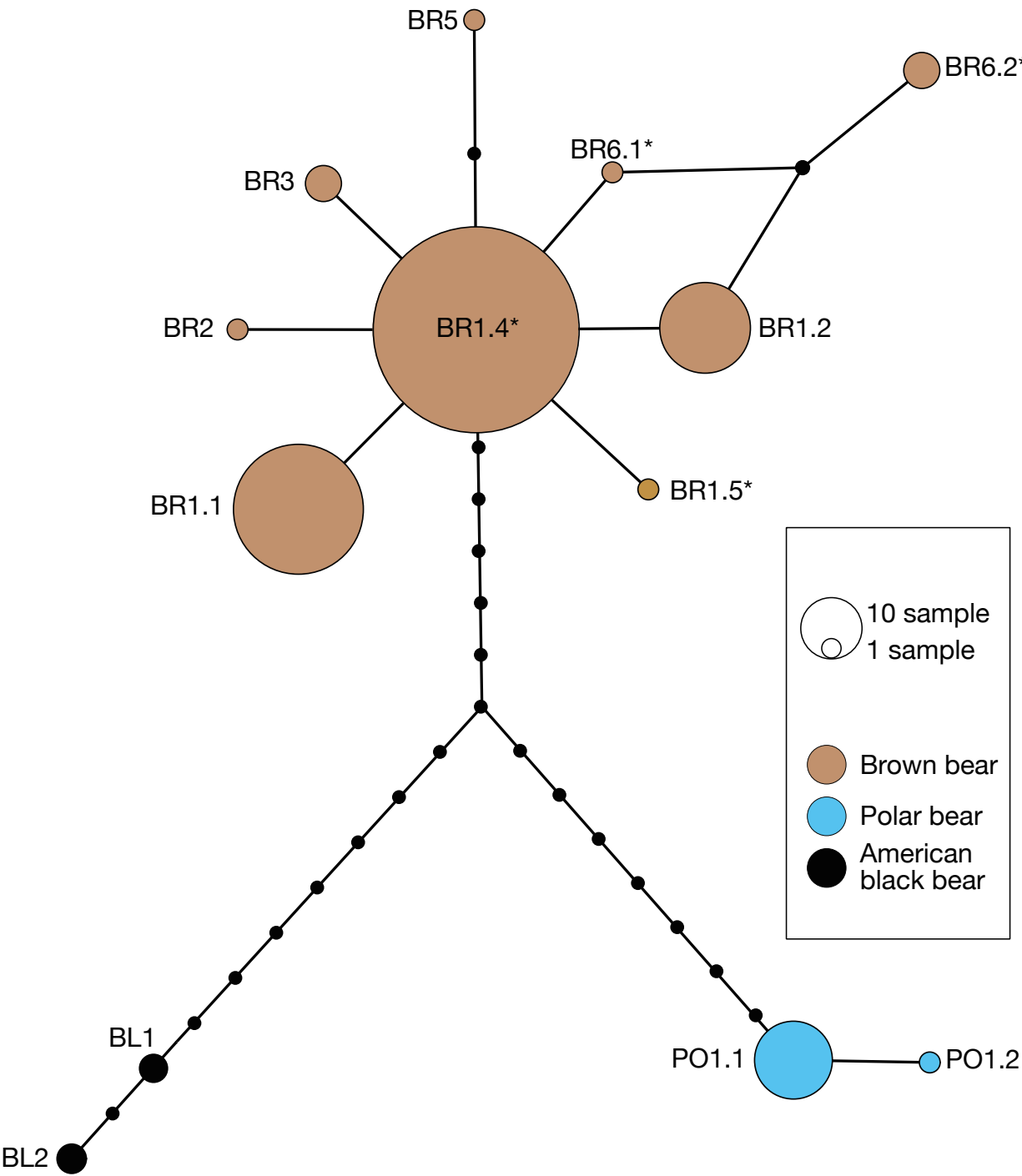

Supplement: Supplementary file 1 — Median-joining haplotype network for brown, polar, and American black bears, based on the 3.1 kb Y-linked data set. Figure S2. Median-joining haplotype network for brown, polar, and American black bears, based on the 5.3 kb Y-linked data set. Figure S3. Median-joining haplotype network for brown bears, based on Y-chromosomal compound haplotypes combined with Y-linked SNPs from a 3.1 kb data set and Y-linked microsatellites. Haplotypes enclosed by a dashed line are from Hokkaido (including one Kunashiri brown bear). Figure S4. Map of Eurasia showing the geographical distribution of brown bear Y-chromosomal compound haplotypes. Each symbol represents one individual. Figure S5. Enlargement of the larger boxed area in Fig. S4, showing the geographical distribution of brown bear Y-chromosomal compound haplotypes around the Ural Mountains. (ZIP 4.95 mb) [file 40851_2017_84_MOESM1_ESM.zip › 170811 FigS2.pdf]

Fig. S1

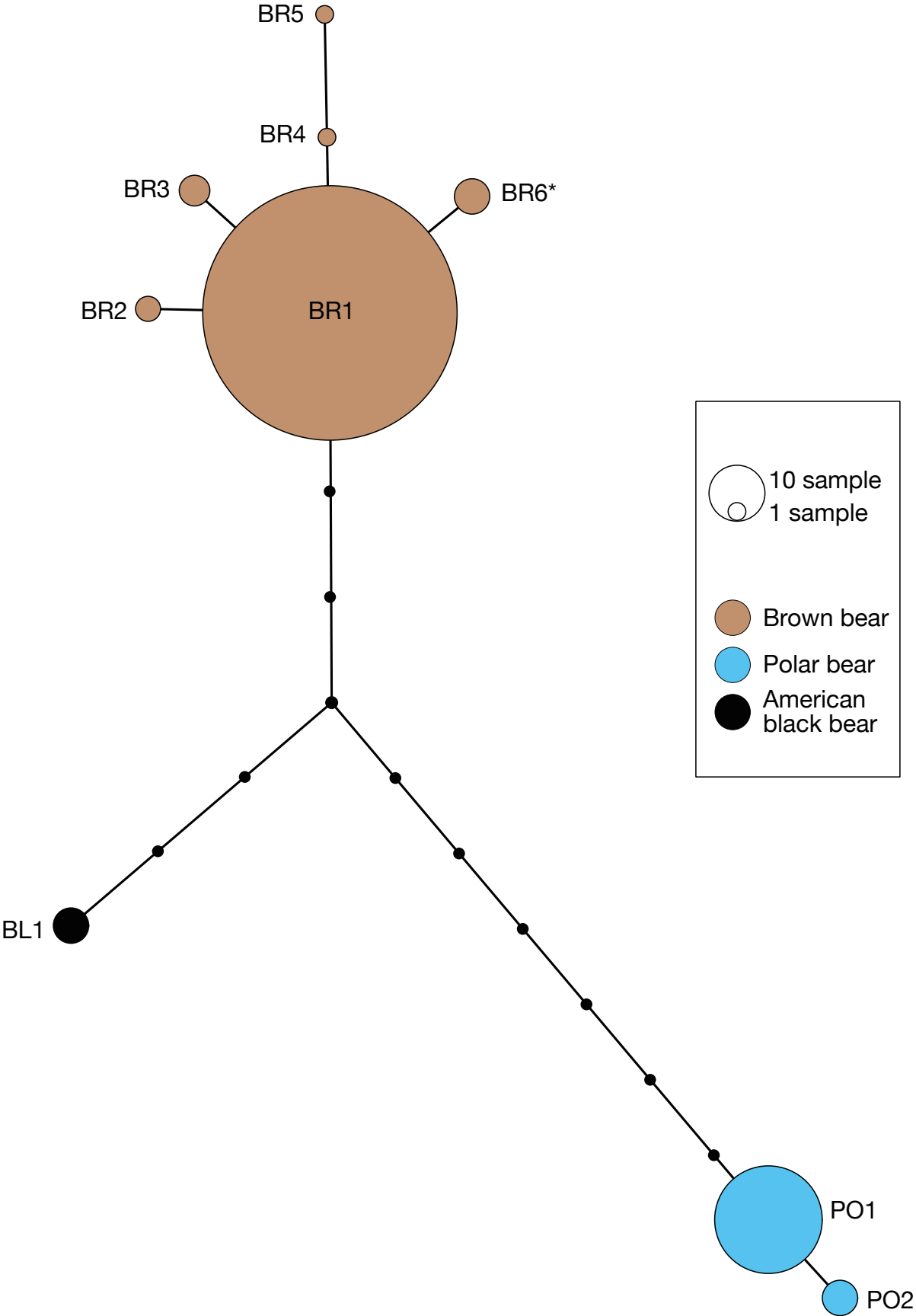

Supplement: Supplementary file 1 — Median-joining haplotype network for brown, polar, and American black bears, based on the 3.1 kb Y-linked data set. Figure S2. Median-joining haplotype network for brown, polar, and American black bears, based on the 5.3 kb Y-linked data set. Figure S3. Median-joining haplotype network for brown bears, based on Y-chromosomal compound haplotypes combined with Y-linked SNPs from a 3.1 kb data set and Y-linked microsatellites. Haplotypes enclosed by a dashed line are from Hokkaido (including one Kunashiri brown bear). Figure S4. Map of Eurasia showing the geographical distribution of brown bear Y-chromosomal compound haplotypes. Each symbol represents one individual. Figure S5. Enlargement of the larger boxed area in Fig. S4, showing the geographical distribution of brown bear Y-chromosomal compound haplotypes around the Ural Mountains. (ZIP 4.95 mb) [file 40851_2017_84_MOESM1_ESM.zip › 170811 FigS1.pdf]

Fig. S5

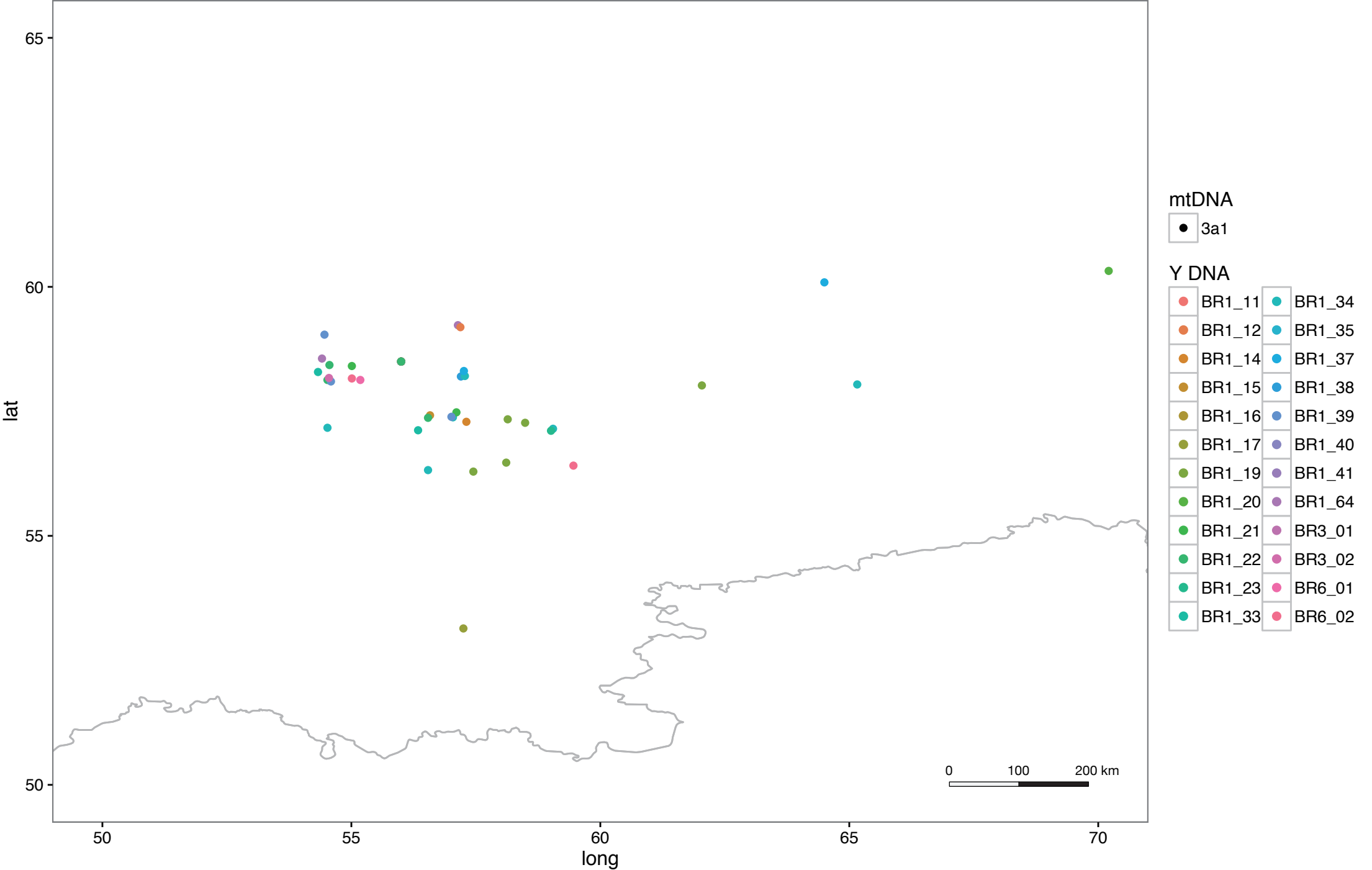

Supplement: Supplementary file 1 — Median-joining haplotype network for brown, polar, and American black bears, based on the 3.1 kb Y-linked data set. Figure S2. Median-joining haplotype network for brown, polar, and American black bears, based on the 5.3 kb Y-linked data set. Figure S3. Median-joining haplotype network for brown bears, based on Y-chromosomal compound haplotypes combined with Y-linked SNPs from a 3.1 kb data set and Y-linked microsatellites. Haplotypes enclosed by a dashed line are from Hokkaido (including one Kunashiri brown bear). Figure S4. Map of Eurasia showing the geographical distribution of brown bear Y-chromosomal compound haplotypes. Each symbol represents one individual. Figure S5. Enlargement of the larger boxed area in Fig. S4, showing the geographical distribution of brown bear Y-chromosomal compound haplotypes around the Ural Mountains. (ZIP 4.95 mb) [file 40851_2017_84_MOESM1_ESM.zip › 170811 FigS5.pdf]
